# Working Memory Training for Healthy Older Adults: The Role of Individual Characteristics in Explaining Short- and Long-Term Gains

**DOI:** 10.3389/fnhum.2017.00099

**Published:** 2017-03-22

**Authors:** Erika Borella, Elena Carbone, Massimiliano Pastore, Rossana De Beni, Barbara Carretti

**Affiliations:** ^1^Department of General Psychology, University of PadovaPadova, Italy; ^2^Department of Developmental and Social Psychology, University of PadovaPadova, Italy

**Keywords:** working memory training, older adults, age, working memory baseline performance, general cognitive ability, training benefits, individual differences, individual characteristics

## Abstract

**Objective:** The aim of the present study was to explore whether individual characteristics such as age, education, vocabulary, and baseline performance in a working memory (WM) task—similar to the one used in the training (criterion task)—predict the short- and long-term specific gains and transfer effects of a verbal WM training for older adults.

**Method:** Four studies that adopted the Borella et al. (2010) verbal WM training procedure were found eligible for our analysis as they included: healthy older adults who attended either the training sessions (WM training group), or alternative activities (active control group); the same measures for assessing specific gains (on the criterion WM task), and transfer effects (nearest on a visuo-spatial WM task, near on short-term memory tasks and far on a measure of fluid intelligence, a measure of processing speed and two inhibitory measures); and a follow-up session.

**Results:** Linear mixed models confirmed the overall efficacy of the training, in the short-term at least, and some maintenance effects. In the trained group, the individual characteristics considered were found to contribute (albeit only modestly in some cases) to explaining the effects of the training.

**Conclusions:** Overall, our findings suggest the importance of taking individual characteristics and individual differences into account when examining WM training gains in older adults.

## Introduction

Working memory (WM), i.e., the ability to retain and manipulate information for use in complex cognitive tasks, is one of the core mechanisms involved in higher-order cognitive abilities (e.g., fluid intelligence, problem-solving, and reading comprehension; de Ribaupierre, 2001; Borella et al., 2011). Though characterized by a limited capacity, WM is a crucial mechanism in cognition. It is also one of the cognitive processes that suffer a clear and linear decline with aging (e.g., Borella et al., 2008; Mammarella et al., 2013). WM is consequently one of the general processes targeted by the new generation of process-based cognitive training. The assumption that WM is trainable is based on evidence of the plasticity of our cognitive system across the whole life span (i.e., Hertzog et al., 2008). Further, according to some WM models, such as the continuity model (see Cornoldi and Vecchi, 2003; Cornoldi, 2010), WM is characterized by different processes that depend on the type of content processed (verbal vs. spatial) and also on the involvement of executive control. Therefore, by improving WM, its related processes can also theoretically be enhanced. The Cornoldi and Vecchi WM model distinguished between a “basic structure” (a sort of personal biological equipment), and a “used ability” determined by the way in which individuals use their WM. On this basis, the benefits of training may presumably concern not only the basic structure of WM, but also its usage.

The aim of WM training in aging is thus to improve older adults' information processing system (e.g., Zinke et al., 2012; Bürki et al., 2014), in order to sustain their cognitive functioning for an active aging. WM training was shown to improve performance not only in the trained tasks (or in tasks similar to the one used in the training), but also in untrained tasks (transfer effects). Training changes the way in which individuals process information, enabling them to make more flexible use of their own resources.

The recent meta-analysis by Karbach and Verhaeghen (2014), focusing on aging, showed that WM training for older people could promote significant gains both in the trained tasks and in other similar tasks (near transfer effects). There also seemed to be some improvements in untrained tasks that shared some cognitive processes with the task used in the training (far transfer effects), though they were usually small in terms of effect size (see Karbach and Verhaeghen, 2014). There have been mixed reports on the matter of the efficacy of WM training in aging (see Table 1), however, making it necessary to identify which factors are involved in giving rise to training benefits. Among the numerous factors to consider, individual characteristics—such as age, general cognitive ability, and baseline cognitive resources—believed to predict the benefits of memory training (e.g., Verhaeghen and Marcoen, 1996) may also have a role as modulators of WM training outcomes (Bürki et al., 2014). Surprisingly, their role has not been the focus of WM training studies as yet.

**Table 1:** **Peer-reviewed studies of the effects of WM training studies involving older adults and the sample's characteristics (i.e., age)**.

	**Age**	**Short-term gains**			**Long-term gains**			
		**Specific**	**Near**	**Far**	**Follow-up**	**Specific**	**Near**	**Far**
**YOUNG ADULTS VS. OLDER ADULTS**								
Li et al., 2008	Young: 20–30	Yes (Y = O)	Yes (Y = O)	No	3 months	Yes (Y > O)	Yes (Y = O)	No
	Old: 70–80							
Dahlin et al., 2008	Young: 23.88 ± 2.52 Old: 68.31 ± 1.69	Yes (Y > O)	Yes (Y > O but in general restricted to young adults)	No	18 months	Yes (Y = O)	No	No
Richmond et al., 2011	Young: 20	Yes (Y = O)	NE	NE	NE			
	Old: 60–80							
Brehmer et al., 2012	Young: 20–30	Yes (Mixed^*^)	Yes (Mixed^*^)	Yes (Mixed^*^)	3 months	Yes (Y = O)	Yes (Y = O)	Yes (Y = O)
	Old: 60–70							
von Bastian et al., 2013	Young: 19–36 Old: 62–77	Yes (Y = O)	Yes (Y = O but minimal for both age groups)	No	NE			
Bürki et al., 2014	Young: 18–38	Yes (Y = O)	Yes (Y = O but mixed^**^)	No	NE			
	Old: 67.98 ± 5.41							
Heinzel et al., 2014	Young: 24–30	Yes (Y > O)	Yes (Y > O)	Yes (Y > O)	NE			
	Old: 61–75							
Salminem et al., 2015	Young: 20–31	Yes (Y > O)	Yes (Y = O)	No	NE			
	Old: 57–77							
Zając-Lamparska and Trempała, 2016	Young: 20–33 Old: 60–85	Yes (Y = O)	Yes (Y = O)	Yes (Y = O)	NE			
**OLDER ADULTS FROM DIFFERENT AGE BRACKETS**								
Buschkuehl et al., 2008	80.00 ± 3.3	Yes	Yes	Yes (Mixed^**^)	12 months	No	No	No
Borella et al., 2010	65–75	Yes	Yes	Yes	8 months	Yes	No	Yes (Mixed^**^)
Richmond et al., 2011	60–80	Yes	Yes (Mixed^**^)	Yes (Mixed^**^)	NE			
Brehmer et al., 2011	60–70	Yes	Yes	Yes (only in a measure of sustained attention)	NE			
Zinke et al., 2012	77–96	Yes	No	No	NE			
Borella et al., 2013	75–87	Yes	No	No	8 months	Yes	No	Yes (only in a measure of inhibition)
Carretti et al., 2013b	65–75	Yes	Yes	Yes	6 months	Yes	Yes	Yes
Zinke et al., 2013	65–95	Yes	Yes (Mixed^**^)	Yes (Mixed^**^)	9 months	Yes	Yes (only in a verbal WM task)	No
Borella et al., 2014	65–84	Yes	Yes (only for young-adults)	Yes (only for young-adults)	8 months	Yes	No	No
Stepankova et al., 2014	65–74	Yes	Yes	Yes	NE			
Xin et al., 2014	60–82	Yes	Yes	Yes	NE			
Cantarella et al., 2017	65–75	Yes	NE	Yes	NE			
Borella et al., 2017	65–75	Yes	No	Yes (only in the processing speed measure)	6 months	Yes	Yes (Mixed^**^)	Yes (only in the processing speed measure)

Y, younger adults; O, older adults; Y > O, greater improvements in young adults than in older adults; Y = O, comparable benefits in young adults and older adults; NE, not examined; WM, working memory; STM, short term memory;

* Mixed, greater improvements in young adults than in older adults in some tasks, but comparable benefits in young and older adults in other tasks;

** *Mixed, significant results only in some of the measures considered*.

Age is one of the crucial factors that may explain whether and to what extent individuals may gain more or less in terms of both specific training gains (in a given trained task) and transfer effects (e.g., von Bastian and Oberauer, 2014). Some WM training studies examined the role of age in explaining the benefits of training by comparing young and older adults, or considering older adults in different age brackets (see also Borella et al., 2014 for a review). Some of the studies that included both young and older adults analyzed how performance changed over the course of the training sessions (Dahlin et al., 2008; Li et al., 2008; Richmond et al., 2011; Brehmer et al., 2012; von Bastian et al., 2013; Bürki et al., 2014). Brehmer et al. (2012), for instance, considered weekly WM performance scores, pooling participants' daily performance in 7 WM training tasks into a single *t*-standardized WM performance score. They found that young adults gained more than older adults from week 1 to 2, but then the two age groups showed comparable improvements from the second week to the end of training, from week 2 to 4. Bürki et al. (2014) found that age-related differences in the performance of young and older adults persisted over 10 training sessions (with greater improvements in the former). Li et al. (2008) found significant improvements for both young and older adults in two trained spatial *n*-back tasks (though the best performance reached by the older adults was still not as good as that of the younger adults). Other studies reported mixed results, however: age-related differences in favor of young adults were found in some of the trained tasks, while improvements were comparable between the two age groups in others.

As concerns specific training gains (i.e., in the criterion tasks), as shown in Table 1, mixed results were found: comparable benefits in young and older adults in tasks strictly similar to those used in the training were obtained in five studies (Li et al., 2008; Richmond et al., 2011; von Bastian et al., 2013; Bürki et al., 2014; Zając-Lamparska and Trempała, 2016); three studies showed greater improvements in young than in older adults (Dahlin et al., 2008; Heinzel et al., 2014; Salminem et al., 2015); one study obtained mixed results with age-related differences for some criterion tasks but not for others (Brehmer et al., 2012); two studies showed that older adults reached the young adults' baseline performance level on the WM criterion tasks immediately after the training-i.e., at the post-test assessment-(Li et al., 2008; Salminem et al., 2015), and one study found that older adults exceeded the young participants' baseline performance in the criterion task.

Similarly, for near as well as far transfer effects (when found), studies found either no differences between the two age groups, or larger effects in young adults than in older adults, or again mixed results (see Table 1). As for any long-term effects, if they were examined, Brehmer et al. (2012), and Dahlin et al. (2008) found a comparable maintenance of specific training gains between young and older adults. Brehmer et al. (2012) also identified the maintenance of both near and far transfer effects in both age groups. Partially in contrast, Li et al. (2008) found larger long-term specific training gains for young adults than for older ones, while the long-term near transfer effects were comparable between the two age groups (see Table 1).

Among the studies focusing only on older adults (see Table 1), the ones that found significant specific training gains and transfer effects, along with their maintenance were those involving young-old participants (from 60 to 74 years old). Studies that included old-old participants (from 75 to 87 years old), and those considering a broad age range (i.e., from 60 to 82) reported mixed findings in terms of specific and transfer training gains in the short term (see Table 1). As for the maintenance effects, some found limited transfer benefits (Borella et al., 2010, 2013, 2017; Zinke et al., 2013), and others found none (Buschkuehl et al., 2008). In one of these studies, older age also emerged as a negative predictor of training gains and at least some transfer effects (Zinke et al., 2013); it is worth noting that the effect sizes for transfer effects in this case were medium to large for tasks assessing near effects, but only small for far transfer effects (see Table 1).

Taken together, the above studies seem to support a negative role of age in determining the benefits of WM training.

Another variable that may influence the efficacy of cognitive training is general cognitive ability, operationalized in some studies with crystallized intelligence, i.e., performance in a vocabulary test (Zinke et al., 2013). This can be considered an index of general cognitive ability (e.g., Baltes, 1987), and a possible moderator of WM training benefits. The only WM training study that considered this variable found, however, that it did not contribute to explaining WM training gains and transfer effects (Zinke et al., 2013).

Individual differences in cognitive resources, such as WM baseline performance, are another factor that may predict training outcomes (see Jaeggi et al., 2014 for evidence in young adults), but only two WM training studies that focused on older adults (aged 77 to 96, Zinke et al., 2012; aged 65 to 80 and over: Zinke et al., 2013) have considered this variable. One study found a negative correlation between specific gains and participants' baseline WM performance, i.e., those with a weaker baseline WM performance gained more in the trained tasks than those whose WM performance was better (Zinke et al., 2012). The other confirmed this association, i.e., the lower the baseline WM performance, the larger the specific gains in the trained tasks (Zinke et al., 2013). In another study by Bürki et al. (2014), the pre-test score obtained in a reasoning measure was considered instead, and the results indicated that the effects of the training were predicted not by this reasoning score, but by age group.

Overall, the pattern of results concerning the role of individual characteristics and individual differences in training-related performance gains and transfer effects is rather mixed. It is also worth noting that, despite the importance of analyzing individual factors when assessing the benefits of WM training, only three studies have so far addressed this issue directly in relation to aging (Zinke et al., 2012, 2013; Bürki et al., 2014).

Hence the present study, the aim of which was to examine the role of individual differences (or individual characteristics) by jointly considering different factors to identify those capable of influencing short- and longer-term training-induced plasticity, measured in terms of both specific training gains and transfer effects. The factors considered as potential mediators of the efficacy of training (e.g., von Bastian and Oberauer, 2014) were demographic characteristics (i.e., age), baseline WM performance (Zinke et al., 2012) and general cognitive ability (i.e., crystallized intelligence measured with a vocabulary test; Zinke et al., 2013). The role of education was also examined because education is considered an index of cognitive efficiency that can preserve cognitive functioning, and because it is also used as a proxy of cognitive reserve (e.g., Stern, 2002; Staff et al., 2004), although no studies have examined whether it interacts with the trainability of WM.

We investigated the role of these variables by analyzing data emerging from studies that adopted the same WM training procedure, developed by Borella et al. (2010). This is one of the few procedures to have been used across different studies, generating consistent and promising results in terms of short- and long-term benefits (Borella et al., 2010, 2017)—also in tasks related to everyday abilities (Carretti et al., 2013b; Cantarella et al., 2017)—in normal and pathological aging (in healthy young-old and old-old, Borella et al., 2013, 2014; in amnestic Mild Cognitive Impairment, Carretti et al., 2013a). The effectiveness of the training has been attributed to the fact that it involves participants practicing with a complex WM span task, combining an adaptive procedure with a systematic variation of the demands of the task, so that it remains constantly novel and challenging, keeping participants interested and motivated during the proposed activities. According to the authors, the training also engages numerous different processes that include encoding, maintaining and inhibiting information, simultaneously managing two tasks, sustaining and shifting attention. Together, these aspects are believed to promote learning and particularly to enable the training to favor transfer effects (see Borella et al., 2010). To date, seven studies have adopted this procedure (see Table 2 for a summary), and four were selected for the present analysis because: (i) the same verbal procedure was adopted; (ii) the same measures were used to assess training gains and transfer effects; (iii) a follow-up session was included; and (iv) a sample of healthy older adults was considered (see Tables 2, 3).

**Table 2:** **Summary of the characteristics and results of the seven studies that employed the Borella et al. (**2010**) training program**.

**Study**	**Training task**	**Months since training: follow-up**	**Older adults' characteristics**	**Specific effects**	**Nearest/near transfer effects**	**Far transfer effects**	**Maintenance effects**
Borella et al., 2010	Verbal WM task (CWMS)	8 months	Healthy 65–75 (*M* = 69 ± 3.18)	Yes	Yes (verbal STM)	Yes (processing speed, inhibition, fluid intelligence)	Yes (verbal WM, processing speed, fluid intelligence)
Carretti et al., 2013b	Verbal WM task (CWMS)	6 months	Healthy 65–75 (*M* = 69 ± 3.60)	Yes	Yes (WM updating)	Yes (comprehension, fluid intelligence)	Yes (all short-term effects)
Borella et al., 2013	Verbal WM task (CWMS)	8 months	Healthy 75–87 (*M* = 79 ± 3.22)	Yes	No	No	Yes (verbal WM, inhibition)
Borella et al., 2017	Verbal WM task (CWMS) ± mental imagery strategy	6 months	Healthy (*M* = 69 ± 4.33)	Yes (all trained groups)	No	Yes (processing speed, more consistent for WM trained group)	Yes (verbal WM, verbal STM, processing speed more consistent for WM trained group)
Carretti et al., 2013a	Verbal WM task (CWMS)	NE	Amnestic MCI 65–75 (*M* = 70 ± 2.40)	Yes	Yes (visuo-spatial WM)	Yes (fluid intelligence)	NE
Borella et al., 2014	Visuo-spatial WM task (The Matrix Task)	8 months	Healthy 65–75 (*M* = 69 ± 2.84), 76–84 (*M* = 80 ± 2.29)	Yes (all age groups)	Yes (verbal WM, visuo-spatial STM, only young-old)	Yes (processing speed, only young-old)	Yes (visuo-spatial WM, verbal WM, all age groups)
Cantarella et al., 2017	Verbal WM task (CWMS)	NE	Healthy 65–75 (*M* = 69 ± 3.08)	Yes	NE	Yes (everyday problems solving, reasoning)	NE

*WM, working memory; STM, short-term memory; MCI, mild cognitive impairment; NE, not examined*.

**Table 3:** **Summary of the sample sizes and outcome measures considered in the four studies conducted using the Borella et al. (**2010**) training program and analyzed in the present study**.

**Study**		**Specific effects**					**Nearest transfer effects**			**Near transfer effects**		**Far transfer effects**				
	***N***	**Trained group**	**Control Group**	**Ed**	**Voc**	**VWM**	**Working memory**			**Short-term memory**		**Comprehension**	**Fluid intelligence**		**Processing speed**	**Inhib**.
						**CWMS**	**Listening span test**	**Dot matrix task**	**Jigsaw puzzle test**	**Updating task**	**Forward and backward digit span task**	**Listening and reading comprehension**	**Cattell test**	**Letter sets**	**Pattern comparison task**	**Stroop color task**
Borella et al., 2010	40	20	20	+	+	+		+			+		+		+	+
Carretti et al., 2013b	36	17	19	+	+	+				+		+	+			
Borella et al., 2013	36	18	18	+	+	+		+			+		+		+	+
Borella et al., 2017^∧^	36	18	18	+	+	+	+		+		+			+	+	
Total Sample	148	73	75	148	148	148	36	76	36	36	112	36	112	36	112	76
Common measures				^*^	^*^	^*^		^*^			^*^		^*^		^*^	^*^

*The measures that the studies had in common are identified in the bottom row of the table with an asterisk. Ed, Education; Voc, Vocabulary Score; VWM, verbal Working Memory; Inhib., inhibition; CWMS, Categorization Working Memory Span Task (De Beni et al., 2008); Listening Span Test (De Beni et al., 2008); Dot Matrix Task (adapted from Miyake et al., 2001); Jigsaw Puzzle Test (De Beni et al., 2008); Updating Task, Working Memory Updating Word Span (Carretti et al., 2010); Forward and Backward Digit Span Task (De Beni et al., 2008); Listening and Reading Comprehension, Listening Comprehension of Spatial Description (Pazzaglia et al., 2007) and Reading Comprehension of Expository Tests (see Borella et al., 2011); Cattell Test, Culture Fair Test, Scale 3 (Cattell and Cattell, 1963); Letter Sets (Ekstrom et al., 1976); Pattern Comparison Test (adapted from Salthouse and Babcock, 1991); Stroop Color Task (adapted from Trenerry et al., 1989)*.

∧ *Only the group that attended the Verbal WM training without the use of the imagery strategy was considered here*.

Specific training gains and transfer effects were categorized along a conceptually-based continuum of nearest to far transfer tasks (i.e., Noack et al., 2009). The complex WM task (the Categorization Working Memory Span task, CWMS) was used to assess specific training gains because it is similar to the task administered to participants during the training sessions. Another complex WM task measuring the same narrow ability (WM), and also involving active processes (see Cornoldi and Vecchi, 2003), but with a different type of material (visuo-spatial, the Dot Matrix Task) was administered to assess what we describe here as *nearest transfer effects*. Measures of the same broad ability (memory), but with different demands from those of the other complex WM tasks (the Forward and Backward Digit Span tests; see meta-analyses by Bopp and Verhaeghen, 2005) were used to assess near transfer effects. Finally, tasks assessing fluid intelligence (the Cattell test), processing speed (the Pattern Comparison task), and inhibitory mechanisms (Stroop Color test and intrusion errors in the CWMS), i.e., mechanisms differing from WM but known to correlate with WM and to help explaining the age-related decline in WM (e.g., de Ribaupierre and Lecerf, 2006), were used to measure far transfer effects.

Linear mixed effects (LME) models were used to examine the role of individual characteristics (demographic variables) and individual differences in predicting improvements in the measures used to assess the effects of the training (in terms of training gains and transfer effects). These models afford a more robust analytical approach for addressing problems associated with hierarchical and correlated data than the traditional analyses generally conducted in training studies (e.g., ANOVA, *t*-test). In particular, LME models allow for a more flexible approach in dealing with individual changes over time when repeated measures are considered (e.g., Gueorguieva and Krystal, 2004; Wainwright et al., 2007; Baayen et al., 2008).

In general, we expected to confirm the beneficial effect of the WM training in terms of short- and long-term gains in the criterion task, and at least short-term transfer effects for all the measures considered. The advantage of performing such an analysis on all four studies sharing the same procedure lay in enabling us to establish the strength of the effects (i.e., effect sizes) on a larger sample.

Concerning the main objective of the study, we used LME models to analyze participants' individual characteristics and differences vis-à-vis the short- and long-term effects of their training. Analyzing these potential predictors will enable us to test the two proposed theoretical explanations for individual differences in training-related performance gains, i.e., a compensation or a magnification effect of process-based training on cognition in older adults (see for example Titz and Karbach, 2014; see also Lövdén et al., 2012). If there is a magnification effect, then individuals who already perform well will benefit the most from the WM training. In other words, high-performing participants may have more efficient cognitive resources and therefore be in a better position to learn and implement new abilities. The WM training should therefore result in a magnification of age-related (in older adults) and individual differences; baseline cognitive performance should also be positively associated with training-related gains and transfer effects. If there is a compensation effect, on the other hand, then high-performing individuals will benefit less from the training because they are already functioning at their optimal level, and thus have less room for improvement. In this case, age-related and individual differences should be reduced after the training, and baseline cognitive performance should be negatively associated with training-induced gains.

The magnification and compensation effects would thus lead in opposite directions. Among the older adults, the younger participants with a good cognitive status, as represented by a measure of crystallized intelligence (vocabulary), a good WM (revealed by their baseline CWMS performance), and a good education might profit more from an adaptive training on a complex aspect of cognition—WM—because a relatively high level of functioning is required to actively engage in and benefit from the activities proposed in the training (Bissig and Lustig, 2007; Lustig et al., 2009), which would, in turns, magnify their abilities. On the other hand, older participants with a worse cognitive status might benefit more from the WM training (Zinke et al., 2012, 2013) because it could counteract the suboptimal use of resources typical of aging by prompting a more flexible use of these resources, more reliant on controlled than on automatic processes, that would re-activate the older participants' potential, having a compensatory effect.

There is also the possibility, as emerges from the results obtained by Zinke et al. (2013), that the factors thought to predict training-related gains might depend on the measures considered, because these factors may also take effect independently. In fact, the individual characteristics examined may explain the training gains differently, as the transfer tasks vary in several aspects—not only in terms of their relationship with WM, but also in terms of the processes involved, such as the type of control (passive as in the short-term memory tasks vs. active as in the reasoning task), or the more or less strong involvement of fluid abilities (stronger in reasoning and in processing speed than in short-term memory tasks) and/or those related to knowledge.

## Method

Table 1 lists the characteristics and the main results of the seven studies that used the verbal WM training procedure developed by Borella et al. (2010). As mentioned in the Introduction, three studies (in the last rows of Table 2) were not considered because: one involved a sample of older adults with mild cognitive impairment (Carretti et al., 2013a); one used a visuo-spatial version of the training program (Borella et al., 2014); and one did not include a follow-up assessment (Cantarella et al., 2017).

The four studies considered eligible for the present analysis had in common: (i) the same verbal procedure; (ii) the same measures for assessing training gains and transfer effects (see Table 3); and (iii) a follow-up assessment.

### Participants

All the four studies considered here included a sample of healthy older adults (all native Italian speakers) recruited from the University of the Third Age, at social clubs in north-eastern Italy, or by word of mouth, who all volunteered for the study.

They were told, either individually (Borella et al., 2010, 2017) or at a plenary session (Borella et al., 2013; Carretti et al., 2013b), that they would be involved in one of two different programs each consisting of five individual sessions, plus a final one at a later date (follow-up). They were also told that the activities proposed in one program would concern their cognitive functioning (i.e., practicing with memory tasks), while in the other one they would be asked to reflect on aspects of memory (e.g., autobiographical recall) and complete some questionnaires.

Depending on the study, participants had to meet the following inclusion criteria: (i) good physical and mental health, assessed by means of a questionnaire and a semi-structured interview, respectively (as in Borella et al., 2010; Carretti et al., 2013b); (ii) none of the exclusion criteria proposed by Crook et al. (1986) as in Borella et al. (2010, 2013); (iii) a Mini-Mental State Score (Folstein et al., 1975) higher than 27 (as in Borella et al., 2013); (iv) a maximum score on the Italian Checklist for Multidimensional Assessments (SVAMA; Gallina et al., 2006), i.e., no signs of incipient dementia (as in Borella et al., 2017). In all four studies, participants were randomly assigned to either the trained group or the active control group.

Overall, 148 participants were involved in the four studies considered, with 73 forming the trained groups, and 75 the active control groups. The pooled trained and control groups were comparable in terms of age (age range: 61–87; trained group: *M* = 71.63, *SD* = 5.53; control group: *M* = 71.61 *SD* = 5.67), *F*_(1, 146)_ <1, years of formal education (from 8 to 24 years; trained group: *M* = 9.42 *SD* = 4.54; control group: *M* = 9.97 *SD* = 4.72), *F*_(1, 146)_ <1, and vocabulary score in the Wechsler Adult Intelligence Scale—Revised (WAIS–R; Wechsler, 1981; max 70; trained group: *M* = 49.21 *SD* = 10.89; control group: *M* = 47.04 *SD* = 11.87), *F*_(1, 146)_ = 1.33, *p* = 0.25.

As common outcome measures used to assess transfer effects varied within the four studies considered, pooled trained and control groups were compared with respect to demographic characteristics and vocabulary score. The pooled trained and control groups were not statistically different in terms of age, years of formal education, and vocabulary score^1^.

## Materials

### Criterion task

#### Categorization Working Memory Span (CWMS) task (De Beni et al., 2008)

The task consisted of 10 sets of word lists, each including 20 lists of words (divided into groups containing from 2 to 6 lists). Participants listened to a set of word lists audio-recorded at a rate of 1 word per second and they had to tap with their hand on the table whenever an animal noun was heard (processing phase). The interval between word lists was 2 s. At the end of a set, participants recalled the last word on each list (maintenance phase)—i.e., they needed to remember from 2 to 6 words altogether, depending on the difficulty of the set.

The total number of words recalled was used as the measure of WM performance (maximum 20).

### Nearest transfer effects

#### Visuo-spatial WM task

##### Dot Matrix task (adapted from Miyake et al., 2001)

In this task participants had to check a matrix equation consisting of an addition or a subtraction presented as lines drawn on a 3 × 3 matrix, and to memorize sequences of dots presented on a 5 × 5 grid. They were given a maximum of 4.5 s to check each equation and say “True” or “False.” Immediately after they gave their answer, they were shown a 5 × 5 grid containing a dot in one of the squares for 3 s. After seeing sets of two to six pairs of equations and grids, they had to indicate the positions of the dots on a blank 5 × 5 grid. There was one practice trial with two equations, each with one dot. The number of dot locations to recall increased from two to six. A total of 28 equations and 28 matrices were presented. The total number of dot positions correctly recalled was considered as the dependent variable (maximum score 14).

### Near transfer effects

#### Short-term memory tasks

##### Forward and Backward Digit Span tasks (De Beni et al., 2008)

Participants had to repeat series of digits in the same (forward) or reverse (backward) order. Each level (from 3 to 9 digits for the forward task, from 2 to 8 digits for the backward task) contained two series of digits. After two consecutive recall errors, the task was discontinued. One point was awarded for each correctly recalled series. The final score corresponded to the total number of series recalled correctly (maximum score of 14 for both tasks).

### Far transfer effects

#### Fluid intelligence

##### Culture Fair test (Cattell test; Cattell and Cattell, 1963)

This task consisted of 4 subtests (to be completed in 2.5–4 min., depending on the subtest) in which participants were asked to: (1) choose from among six different options which ones correctly completed a series of figures; (2) identify figures or shapes that did not belong in a sequence; (3) choose items that correctly completed matrices of abstract figures; (4) assess relationships between sets of items. The dependent variable was the number of correct answers across the four subtests (maximum score 50).

#### Processing speed

##### Pattern comparison task (adpated from Salthouse and Babcock, 1991)

In this task, participants had to decide whether arrangements of line segments were identical or not. The items to be compared were set out on two pages each containing 30 items. Responses consisted of writing *S* (for *Si* [*Yes*], for identical items) or *N* (for *No*, for different items) on the line between the two items in each pair. The experimenter used a stopwatch to record the time taken to complete each page. Three practice trials were run before the experiment started. The dependent variable was the total time taken to complete the task.

#### Inhibition

##### Stroop Color task (adapted from Trenerry et al., 1989)

In this task participants were shown six cards. The first two contained names of colors printed in an incongruent ink color (Incongruent condition); the third and fourth contained names of colors printed in a congruous ink color (Congruent condition); and the last two contained color patches (Control condition). Participants had to name the ink color of each stimulus and were asked to process the stimuli as quickly and accurately as possible. The experimenter recorded response latencies for all conditions by using a stopwatch to time the interval between naming the first and last stimuli—as typically done in other studies using the paper version (e.g., West and Alain, 2000; Van der Elst et al., 2006; Troyer et al., 2006)—and noted the respondents' accuracy by hand on a prepared form. The dependent variable—in order to control for individual differences at the baseline (e.g., Borella et al., 2009)—was the interference index computed in terms of relative difference between the time taken to complete the task in the incongruent and control conditions, that is [(incongruent condition − control condition)/control condition]. A higher score thus implied a greater difficulty in controlling the prepotent response in the incongruent condition.

##### Intrusion errors in the CWMS -CWMS intrusion errors- (De Beni et al., 2008)

The total intrusion errors made in the CWMS, i.e., words that were not actually the last in of each string of words presented, were also considered as a measure of inhibition, representing a participant's ability to inhibit no longer relevant information (Borella et al., 2007).

For each task, two parallel versions were devised and administered in a counterbalanced order across the testing sessions.

### Procedure

Participants attended six individual sessions: the first and fifth were the pre-test and the post-test sessions, and the sixth was for the follow-up (held 6–8 months later). For the other three sessions, the trained participants attended the training program, while the active controls were involved in alternative activities. For both groups, all activities were completed within a 2-week time frame, with a fixed 2-day break between sessions. The duration of the sessions and the amount of interaction with the experimenter were much the same for the two groups.

The three sessions of WM training (sessions 2-3-4) lasted about 30–40 min. Participants were presented with lists of words audio-recorded and organized in the same way as for the CWMS task, and asked to recall target words, and to tap with their hand on the table when they heard an animal noun. The maintenance demand of the CWMS task was manipulated by increasing the number of words that successful participants were asked to recall, and by presenting the lowest memory load to participants who were unsuccessful (session 2). The demands of the task also varied and, depending on the session, they could involve having to recall: (i) the last or first word in each list; (ii) words preceded by a beep sound. The processing demand (tapping on the table when an animal noun occurred) was also manipulated by varying the frequency of the animal words in the lists (session 3). Participants in the active control group were asked to complete questionnaires on memory (session 1: Autobiographical Memory questionnaire; session 2: Memory Sensitivity questionnaire—De Beni et al., 2008), and on psychological well-being (De Beni et al., 2008; see Borella et al., 2010 for more details of the training program and the active control group's activities).

The procedure was completed in accordance with the Declaration of Helsinki (2008).

## Results

### Linear mixed models

Linear mixed effects (LME) models were used to analyze the data because, unlike the more classical and frequently-used methods, they enable estimates to be adjusted for repeat sampling (when more than one observation arises from the same individual), and for sampling imbalance (when some individuals are sampled more than others), and because they allow for variation among individuals within the data (McElreath, 2016). Adopting the Bayesian approach when estimating parameters enabled us to exploit all the advantages of LME modeling, focus directly on the probability of an effect, given the observed data (posterior probability), and compute the evidence of our results.

#### Analytical plan

For each of the eight measures of interest (i.e., the total number of words recalled in the CWMS, the total number of dots recalled in the Dot Matrix task, the total number of series correctly recalled in the Forward and in the Backward Span tasks, the total number of correctly answered items in the Cattell test, the total time taken to complete the task in the Pattern Comparison test, the interference index in the Stroop Color task, and the total number of intrusion errors in the CWMS), we tested several mixed effects models including all combinations of predictors, i.e., group (trained vs. control), age, education, vocabulary, baseline performance in the verbal WM task (the CWMS), and subjects as random effects. More precisely, we started from the null model (i.e., the model with no predictors), considering only the longitudinal effect (pre-test, post-test, follow-up) and subsequently introduced all the predictors and the interactions of all the predictors with the sessions.

For data analysis, we proceeded as follows:

- A first graphical inspection of the univariate and bivariate distributions of all the outcome variables considered, then their summarizing with descriptive statistics (see Table 4). This step was done to: (i) check the data distributions and identify any errors/anomalies (e.g., wrong label codes or potential outliers); and to (ii) ascertain which model to adopt for a better fit of our data and choose appropriate priors for the parameters.
- Model fitting and parameter estimation: each model was fitted (separately for each measure of interest) using the Bayesian MCMC estimation method implemented in the STAN probabilistic programming language (Stan Development Team, 2015) with the R packages rstanarm (Gabry and Goodrich, 2016) and brms (Buerkner, 2016).
  For the regression parameters (ß) we used normal priors (M = 0 and *SD* = 10), and for standard deviation parameters we used half-Student *t* (*df* = 3, *M* = 0, *SD* = 10); convergences were assessed by examining the potential scale reduction factor (PSRF; Gelman and Rubin, 1992).
- Comparison between the models (separately for each measure of interest) to identify the best one. We considered the Widely Applicable Information Criterion (WAIC; Watanabe, 2010), where lower values indicate a better fit, and the Akaike Weight, i.e., an estimate of the likelihood of a model making the best prediction on new data, conditional on the set of models considered (Burnham et al., 2011; McElreath, 2016).
- Analysis of the best model using posterior distributions of parameters. Parameter estimates were summarized by using posterior means and 95% Credibility Intervals (CI; Kruschke, 2011).

**Table 4:** **Descriptive statistics for the outcome measures by group (trained vs. controls) and by assessment session (pre-test, post-test, follow-up)**.

	**Trained group**										**Active control group**									
		**Pre-test**			**Post-test**			**Follow-up**			**Pre-test**				**Post-test**			**Follow-up**		
	***N***	***M***	***SD***	**Min–Max**	***M***	**SD**	**Min—Max**	***M***	***SD***	**Min–Max**	***N***	***M***	**SD**	**Min–Max**	***M***	***SD***	**Min–Max**	***M***	***SD***	**Min–Max**
CWMS	73	10.23	3.82	4–19	14.68	3.32	6–20	14.05	3.34	6–20	75	10.04	4.30	3–20	10.41	4.18	3–20	10.17	4.20	1–19
Dot Matrix Task	38	5.13	2.18	1–10	7.45	2.84	2–12	5.47	2.02	2–11	38	4.92	2.54	1–12	5.16	2.26	1–12	5.00	2.63	0–10
Forward Span	56	6.21	1.75	2–11	7.18	1.64	3–11	6.46	1.58	3–13	56	6.04	1.63	3–11	5.84	1.45	2–9	5.82	1.40	3–10
Backward Span	56	5.43	1.61	2–10	6.45	1.72	3–10	5.66	1.46	3–11	56	5.02	1.67	2–10	4.84	1.39	2–8	5.12	1.28	2–8
Cattell Test	55	15.13	4.71	6–24	18.87	5.14	7–28	17.9	5.03	7–32	57	15.60	5.46	5–29	16.23	5.32	7–27	15.91	5.49	5–28
Pattern Comparison Test	56	155.15	50.53	59.43–270	127.12	35.67	56.50–229	135.98	33.54	70.12–251	56	165.48	45.87	66.28–261	162.64	46.49	67.40–289	161.28	46.85	59.32–267
Stroop Color index on RTs	38	0.92	0.45	0.08–2.35	0.86	0.44	0.13–1.83	0.94	0.37	0.28–2.03	38	0.95	0.50	0.20–1.89	0.95	0.39	0.40–2.01	0.87	0.47	−0.03 to 1.75
CWMS intrusions	73	1.95	1.88	0–8	1.14	1.65	0–7	0.97	1.28	0–7	75	1.77	2.15	0–8	1.59	1.73	0–8	1.84	2.09	0–9

*CWMS, Categorization Working Memory Span Task; RTs, response times; CWMS intrusions, intrusion errors in the CWMS*.

#### Data inspection

Table 3 contains the descriptive statistics for each of the measures of interest by group (trained and control), and by assessment session (pre-test, post-test, and follow-up).

#### Model fitting and parameter estimation

In all, 1,026 models, 58 for the CWMS, and 121 for each of the other measures of interest, were fitted. The fit indices of the 5 best models for each measure of interest are given in Table 5.

**Table 5:** **Fit indices of the five best models for each variable of interest**.

**Measure of interest**		**Model**	**Formula**	**WAIC**	**^δ^_WAIC_**	**weight**
CWMS	1	M236	ss × group × vocab	1912.49	0.00	0.59
	2	M313	ss × age + ss × group + ss × vocab	1914.53	2.03	0.21
	3	M216	ss × group + ss × vocab	1916.38	3.88	0.08
	4	M333	ss × age × group × vocab	1917.19	4.69	0.05
	5	M311	ss × age + ss × educ + ss × group	1920.38	7.89	0.01
Dot Matrix task	1	M336	ss × age × group × vocab	879.12	0.00	0.84
	2	M316	ss × age + ss × group + ss × vocab	884.74	5.62	0.05
	3	M233	ss × age × group	885.14	6.02	0.04
	4	M413	ss × age + ss × CWMS baseline + ss × group + ss × vocab	886.33	7.21	0.02
	5	M213	ss × age + ss × group	887.01	7.89	0.01
Forward Span task	1	M340	ss × educ × group × vocab	985.33	0.00	0.27
	2	M339	ss × CWMS baseline × group × vocab	985.63	0.29	0.23
	3	M336	ss × age × group × vocab	986.19	0.85	0.17
	4	M220	ss × group + ss × vocab	988.06	2.72	0.06
	5	M316	ss × age + ss × group + ss × vocab	988.13	2.79	0.06
Backward Span task	1	M334	ss × age × educ × group	1023.03	0.00	0.48
	2	M531	ss × age × educ × vocab × group × CWMS baseline	1024.05	1.01	0.29
	3	M336	ss × age × group × vocab	1025.06	2.02	0.17
	4	M431	ss × age × CWMS baseline × educ × group	1028.94	5.90	0.02
	5	M433	ss × age × CWMS baseline × group × vocab	1029.49	6.45	0.01
Cattell Test	1	M332	ss × age × CWMS baseline × group	1747.97	0.00	0.43
	2	M233	ss × age × group	1749.88	1.90	0.16
	3	M334	ss × age × educ × group	1750.31	2.33	0.13
	4	M434	ss × age × educ × group × vocab	1751.08	3.10	0.09
	5	M314	ss × age + ss × educ + ss × group	1751.45	3.47	0.07
Pattern Comp. Task	1	M339	ss × CWMS baseline × group × vocab	3132.28	0.00	0.38
	2	M233	ss × age × group	3133.55	1.26	0.20
	3	M114	ss × group	3135.60	3.31	0.07
	4	M433	ss × age × CWMS baseline × group × vocab	3135.65	3.36	0.07
	5	M332	ss × age × CWMS baseline × group	3136.00	3.71	0.05
Stroop color index on RTs	1	M233	ss × age × group	174.18	0.00	0.22
	2	M020	Age	176.69	2.51	0.06
	3	M111	ss × age	176.89	2.71	0.05
	4	M060	CWMS baseline	177.35	3.17	0.04
	5	M000	(1 | subject)	177.37	3.18	0.04
CWMS intrusions	1	M332	ss × age × CWMS baseline × group	1481.79	0.00	0.93
	2	M431	ss × age × CWMS baseline × educ × group	1487.59	5.79	0.05
	3	M337	ss × CWMS baseline × educ × group	1491.27	9.48	0.01
	4	M433	ss × age × CWMS baseline × group × vocab	1492.32	10.53	0.00
	5	M531	ss × age × educ × vocab × group × CWMS baseline	1493.49	11.70	0.00

*CWMS, Categorization Working Memory Span Task; ss, session; vocab, vocabulary; educ, education; CWMS baseline, baseline performance level; in the Categorization Working Memory Span Task; CWMS intrusions, intrusion errors in the CWMS; WAIC, Widely Applicable Information Criterion (lower values indicate better fit; Watanabe, 2010); δ_WAIC_, Widely Applicable Information Criterion difference between the best model and the others; weigth, Akaike Weight, i.e., an estimate of the probability that the model will make the best prediction on new data conditional on the set of models considered (Burnham et al., 2011; McElreath, 2016)*.

#### Model comparison and best model analysis

**Figure 1:**
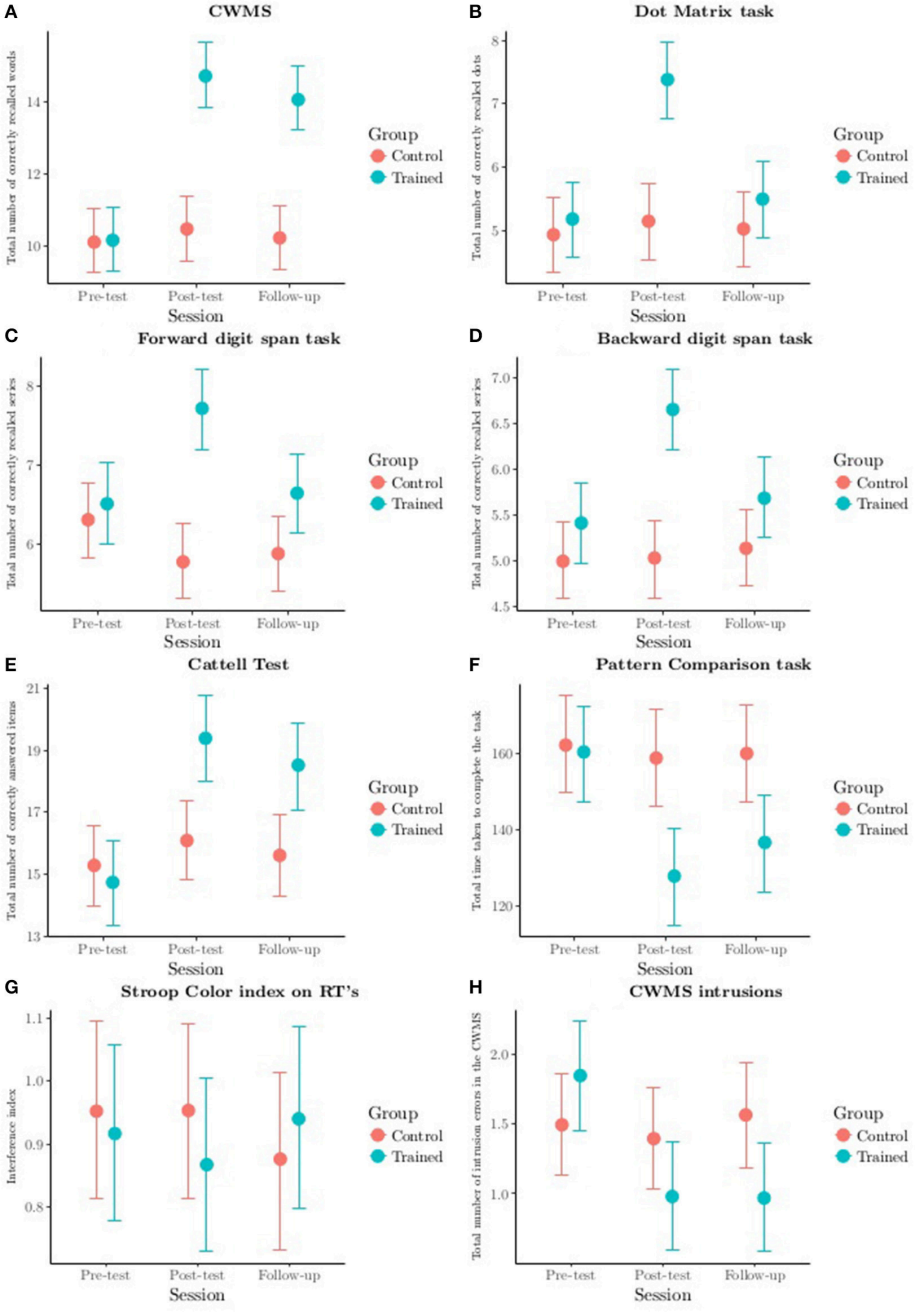
**Group (trained vs. control) X Session (pre-test vs. post-test vs. follow-up) interaction from the best model for each measure of interest**. Categorization Working Memory Span task **(A)**, Dot Matrix task **(B)**, Forward **(C)** and Backward **(D)** digit span tasks, Cattell test **(E)**, Pattern Comparison task **(F)**, Stroop Color task **(G)** and intrusion errors in the Categorization Working Memory Span task **(H)**. CWMS, Categorization Working Memory Span Task; CWMS intrusions, intrusion errors in the Categorization Working Memory Span Task; RTs, Response Times. Segments represent the 95% credibility intervals.

For each outcome measure we compared the fit indices of the 5 best models. Then, focusing on the best one (the one with the lowest WAIC), we proceeded with a graphical inspection of the Group (trained vs. control) X Session (pre-test vs. post-test vs. follow-up) interaction to assess the effectiveness of the training (see Figure 1).

To gain a better understanding of the extent of the benefits of training on the trained group's performance, the effect size was computed on the differences between the two groups (trained and control) at pre-test, post-test, and follow-up (see Table 6). In addition, to ascertain the dimension of the immediate (pre- vs. post-test) and long-term (pre-test vs. follow-up) gains obtained in the trained group, Cohen's d was computed using the following formula: {(Post-test or follow-up for the trained group − Pre-test for the trained group) − (Post-test or follow-up for the controls − Pre-test for the controls)}/(Pooled *SD* of the difference; see Weisz and Hawley, 2001). This enabled us to adjust the gains shown by the trained group in relation to the gain obtained by the active control group (see Table 6).

**Table 6:** **Effect sizes**.

	***d*** **of Cohen**			**Net effect sizes**	
	**Trained group—Active control group**			**Trained group vs. Active control group**	
	**Pre-test**	**Post-test**	**Follow-up**	Short-term gains^*^	Long-term gains^**^
CWMS	0.05	1.13	1.02	1.48	1.54
Dot Matrix Task	0.09	0.89	0.20	0.95	0.16
Forward Digit Span	0.10	0.87	0.43	0.91	0.39
Backward Digit Span	0.25	1.03	0.39	0.80	0.10
Cattell Test	−0.09	0.50	0.39	0.67	0.78
Pattern Comparison Test	−0.21	−0.86	−0.62	−0.72	−0.47
Stroop Color index on RTs	−0.07	−0.22	0.15	−0.12	0.23
CWMS intrusions	0.09	−0.27	−0.50	−0.36	−0.66

*Cohen's d of the difference between the trained and control groups at pre-test, post-test, and follow-up, for each measure of interest (first three columns); and net effect sizes computed immediately after the training (pre-test vs. post-test; short-term gains), then 6/8 months later (pre-test vs. follow-up; long-term gains) for all measures of interest (last two columns). CWMS, Categorization Working Memory Span Task; CWMS intrusions, intrusion errors in the CWMS; RTs, Response Times*.

* *For short-term gains, the net effect size was computed with the formula {(Post-test for the training groups − Pre-test for the training groups) − (Post-test for the controls − Pre-test for the controls)}/(Pooled SD of the difference)*.

** *For the long-term gains, the net effect size was computed with the formula {(Follow-up for the training groups − Pre-test for the training groups) − (Follow-up for the controls − Pre-test for the controls)}/(Pooled SD of the difference)*.

**Figure 2:**
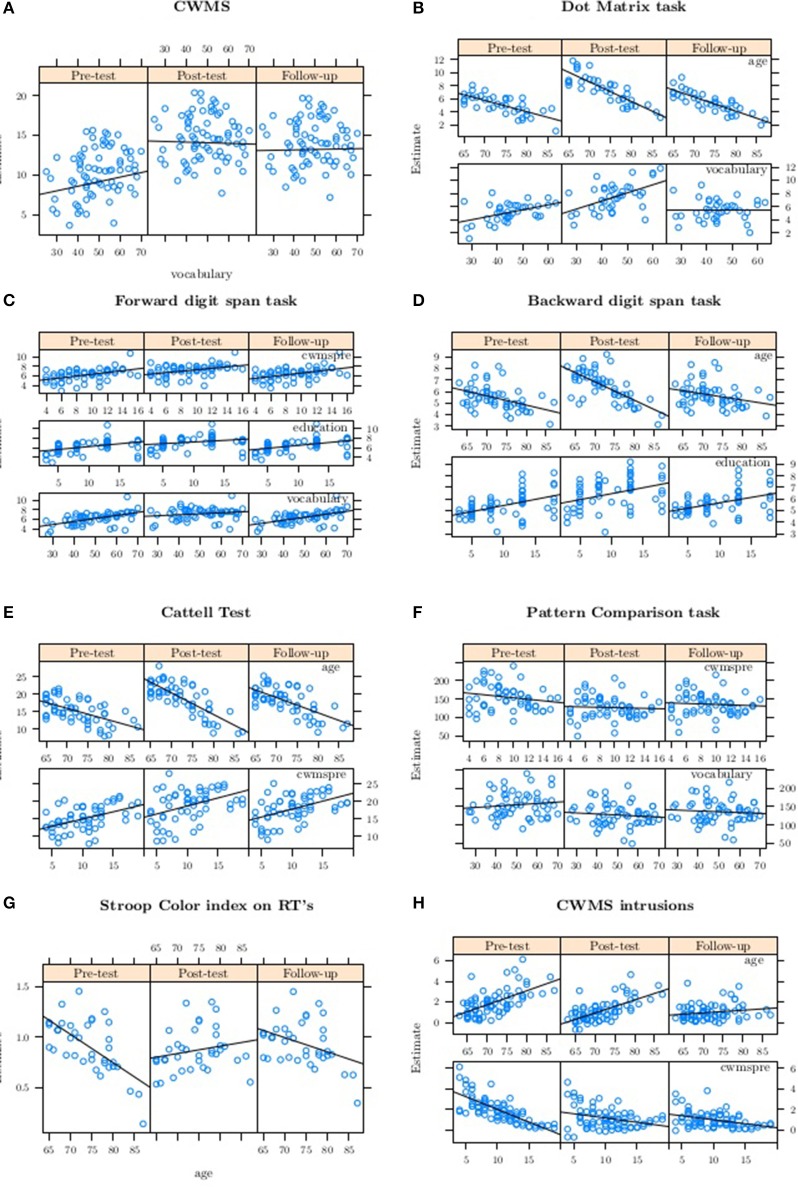
**Fitted values from the best model for the trained group alone for each variable of interest**. Categorization Working Memory Span task **(A)**, Dot Matrix task **(B)**, Forward **(C)** and Backward **(D)** digit span tasks, Cattell test **(E)**, Pattern Comparison task **(F)**, Stroop Color task **(G)** and intrusion errors in the Categorization Working Memory Span task **(H)**. CWMS, Categorization Working Memory Span Task; CWMS intrusions, intrusion errors in the Categorization Working Memory Span Task; RTs, Response Times; cwmspre, CWMS baseline performance level.

Then, for the trained group, we conducted a graphical inspection (separately for each session) of the values fitted for the significant effects of the best model (see Figure 2), supported by the evidence ratio for the hypothesis involving the ß coefficients considered (see Table 7).

**Table 7:** **Evidence ratio of the differences between pre- and post-test slopes, and between post-test and follow-up slopes, for each variable of interest, by fit index**.

**Measure of interest**	**Fitted value**	**Targeted hypothesis**	**Alternative hypothesis**	**Estimate**	**Estimate error**	**Evidence ratio**
CWMS	Vocabulary	ß*_0_ > ß_1_*	ß*_0_ < ß_1_*	0.08	0.04	89.91
		ß*_1_ < ß_2_*	ß*_1_ > ß_2_*	−0.02	0.04	2.86
Dot Matrix task	Age	ß*_0_ > ß_1_*	ß*_0_ < ß_1_*	0.13	0.07	32.06
		ß*_1_ < ß_2_*	ß*_1_ > ß_2_*	−0.08	0.07	7.05
	Vocabulary	ß*_0_ < ß_1_*	ß*_0_ > ß_1_*	−0.04	0.05	5.02
		ß*_1_ > ß_2_*	ß*_1_ < ß_2_*	0.12	0.05	306.69
Forward Span task	Education	ß*_0_ > ß_1_*	ß*_0_ < ß_1_*	0.05	0.04	7.39
		ß*_1_ < ß_2_*	ß*_1_ > ß_2_*	−0.05	0.04	8.83
	Vocabulary	ß*_0_ > ß_1_*	ß*_0_ < ß_1_*	0.04	0.02	199
		ß*_1_ < ß_2_*	ß*_1_ > ß_2_*	−0.04	0.02	199
	CWMS baseline	ß*_0_ > ß_1_*	ß*_0_ < ß_1_*	−0.02	0.06	0.63
		ß*_1_ > ß_2_*	ß*_1_ < ß_2_*	−0.02	0.06	0.67
Backward Span task	Education	ß*_0_ > ß_1_*	ß*_0_ < ß_1_*	0.00	0.05	0.97
		ß*_1_ < ß_2_*	ß*_1_ > ß_2_*	0.01	0.05	0.67
	Age	ß*_0_ > ß_1_*	ß*_0_ < ß_1_*	0.08	0.04	77.43
		ß*_1_ < ß_2_*	ß*_1_ > ß_2_*	−0.11	0.03	665.67
Cattell Test	Age	ß*_0_ > ß_1_*	ß*_0_ < ß_1_*	0.28	0.10	433.44
		ß*_1_ < ß_2_*	ß*_1_ > ß_2_*	−0.18	0.09	41.11
	CWMS baseline	ß*_0_ > ß_1_*	ß*_0_ < ß_1_*	−0.04	0.14	0.61
		ß*_1_ < ß_2_*	ß*_1_ > ß_2_*	0.00	0.15	0.99
Pattern Comparison Task	Vocabulary	ß*_0_ > ß_1_*	ß*_0_ < ß_1_*	0.65	0.45	13.13
		ß*_1_ < ß_2_*	ß*_1_ > ß_2_*	−0.07	0.45	1.25
	CWMS baseline	ß*_0_ < ß_1_*	ß*_0_ > ß_1_*	−1.63	1.58	5.77
		ß*_1_ > ß_2_*	ß*_1_ < ß_2_*	0.19	1.63	1.21
Stroop color index on RTs	Age	ß*_0_ < ß_1_*	ß*_0_ > ß_1_*	−0.04	0.01	665.67
		ß*_1_ > ß_2_*	ß*_1_ < ß_2_*	0.02	0.01	27.99
CWMS intrusions	Age	ß*_0_ > ß_1_*	ß*_0_ < ß_1_*	0.01	−0.05	1.49
		ß*_1_ > ß_2_*	ß*_1_ < ß_2_*	0.1	0.04	189.48
	CWMS baseline	ß*_0_ < ß_1_*	ß*_0_ > ß_1_*	−0.16	0.06	799
		ß*_1_ < ß_2_*	ß*_1_ > ß_2_*	0	0.06	1.14

*CWMS, Categorization Working Memory Span Task; RTs, Response Times; CWMS intrusions, intrusion errors in the CWMS*.

The evidence ratio represents the evidence of the targeted hypothesis (e.g., ß > 0) with respect to the opposite hypothesis (e.g., ß < 0). If the evidence ratio equals 1, then the two hypotheses are equally plausible. An evidence ratio larger than 1 indicates that the target hypothesis is more plausible than the opposite one, and an evidence ratio of <1 means that the opposite hypothesis is more plausible than the targeted one. In the present study, the evidence ratio was used to assess the differences between the pre- and post-test slopes, and between the post-test and follow-up slopes.

### Criterion task

#### CWMS

For the CWMS we considered a total of 58 models^2^. The best model was the one with the Session X Group X Vocabulary interaction, with a probability of 59.1% (see Table 5).

Figure 1A shows the Group (trained vs. control) X Session (pre-test vs. post-test vs. follow-up) interaction from the best model: the trained group performed better at post-test than at pre-test, and maintained its better performance from post-test to follow-up. The effect sizes for group differences were large at both post-test and follow-up (see Table 6). No differences were found for the active control group. The trained group outperformed the control group at post-test and follow-up (see Figure 1A). The net effect size index for the trained group, adjusted on the control group's performance, was large for immediate gains (pre- vs. post-test) and for long-terms gains (see Table 6).

Figure 2A shows the fitted values from the best model for the trained group alone, as a function of vocabulary score at the three assessment sessions, with the relative estimated linear trend. The regression slope decreased from session 0 (pre-test) to session 1 (post-test), with an evidence ratio of 89.91 (see Table 7), and it became flat at follow-up, as shown by the evidence ratio (see Table 7). These results indicate that participants with low vocabulary scores were the ones who showed an improvement in performance in the criterion WM task from pre-test to post-test, and maintained this gain at follow-up.

### Nearest transfer effects

#### Visuo-spatial working memory

##### Dot Matrix task

For the Dot Matrix task we considered a total of 121 models. The best model was given by the Session X Age X Group X Vocabulary interaction with a probability of 84.7%. This model was about 17 times more evident than the next one, which achieved a probability of around 5.1% (see Table 5).

As for the Group (trained vs. control) X Session (pre-test vs. post-test vs. follow-up) interaction, the two groups did not differ at pre-test. The trained group performed better at post-test than at pre-test, but this gain was not maintained at follow-up, when performance was not as good as at post-test (see Figure 1B). Effect sizes for group differences were large at post-test and became small at follow-up (see Table 6). No differences were found for the control group. The trained group only outperformed the control group at post-test (see Figure 1B). The net effect size for the trained group, adjusted on the control group's performance, was large for the immediate gains (pre- vs. post-test), but became small for the long-terms gains (see Table 6).

Figure 2B shows the values fitted from the best model for the trained group alone, as a function of age, and of vocabulary score at the three assessment sessions, with the corresponding estimated linear trend. For age, the regression slope suggests a change—with an evidence ratio of 32.06 (see Table 7)—from session 0 (pre-test) to session 1 (post-test), and a slight deterioration from post-test to follow-up (evidence ratio of 7.05; see Table 7): it was the younger participants whose performance improved from pre-test to post-test, and then dropped back at follow-up to much the same as their pre-test performance.

As for vocabulary, the regression slope rose from session 0 (pre-test) to session 1 (post-test), with an evidence ratio of 5.02 (see Table 7), and clearly dropped again, becoming flat at follow-up (see evidence ratio): it was the participants with high vocabulary scores whose performance improved in terms of the number of dots correctly recalled from pre-test to post-test, but not at follow-up when their performance clearly deteriorated.

### Near transfer effects

#### Short-term memory

##### Forward Digit Span task

A total of 121 models, one of which did not converge, were considered for the Forward Digit Span task. The best model was represented by the Session X Education X Group X Vocabulary interaction with a probability of 27.3%. This model did not seem much more evident than the next two, for which the probability was around 23.5 and 17.7%, respectively (see Table 4).

As for the Group (trained vs. control) X Session (pre-test vs. post-test vs. follow-up) interaction, the two groups did not differ at pre-test. The trained group performed better at post-test than at pre-test, but this gain was not maintained at follow-up, when performance was worse than at post-test (see Figure 1C). The effect sizes for group differences were large at post-test and became small at follow-up (see Table 6). No differences were seen for the control group. The trained group only outperformed the control group at post-test (see Figure 1C). The net effect size for the trained group, adjusted on the control group's performance, was large for immediate gains (pre- vs. post-test), but became small for long-terms gains (see Table 6).

Figure 2C shows the values fitted from the best model for the trained group alone as a function of education, vocabulary, and pre-test performance in the WM criterion task, at the three assessment sessions, with the corresponding estimated linear trend. A minimal change from pre-test to post-test emerged for all the variables: the evidence ratio between session 0 (pre-test) and session 1 (post-test) was 7.39 for education, 199 for vocabulary, and 0.63 for pre-test performance in the WM criterion task (see Table 7). At follow-up, there was a change from post-test (see evidence ratio), with performance dropping back to pre-test levels. In particular, it was the participants who had a limited education, low vocabulary scores, and a poor pre-test performance in the WM criterion task who experienced a slight improvement in their performance, but only at post-test.

##### Backward digit span task

For the Backward Digit Span task we considered a total of 121 models, one of which did not converge. The best model emerged for the Session X Age X Education X Group interaction, with a probability of 48.3%. This model was about two times more evident than the next one, for which the probability was around 29% (see Table 5).

For the Group (trained vs. control) X Session (pre-test vs. post-test vs. follow-up) interaction, the two groups did not differ at pre-test. The trained group performed better at post-test than at pre-test, and then its performance deteriorated from post-test to follow-up (see Figure 1D). The effect sizes for group differences were large at post-test and became small at follow-up (see Table 6). No differences were identified in the control group. The trained group only outperformed the control group at post-test (see Figure 1D). The net effect size for the trained group, adjusted on the control group's performance, was large for immediate gains (pre- vs. post-test), but became small for long-terms gains (see Table 6).

Figure 2D shows the values fitted from the best model for the trained group alone as a function of education, and of age at the three assessment sessions, with the corresponding estimated linear trend. For education, there was no change in the slope. For age, the younger the participants, the greater the improvement in performance from pre-test to post-test, with an evidence ratio of 77.43 (see Table 7). From post-test to follow-up, there was a decline in the slope, with an evidence ratio of 665.67 (see Table 7), meaning that performance returned to the levels seen at pre-test.

### Far transfer effects

#### Fluid intelligence

##### Cattell test

For the Cattell test we considered a total of 121 models. The best model was obtained with the Session X Age X CWMS baseline X Group interaction, with a probability of 43.1%. This model was about three times more evident than the next, for which the probability was around 16.6% (see Table 5).

For the Group (trained vs. control) X Session (pre-test vs. post-test vs. follow-up) interaction, the two groups did not differ at pre-test. The trained group performed better at post-test than at pre-test, and then its performance declined from post-test to follow-up (see Figure 1E). The effect sizes for group differences were medium at post-test and became small at follow-up (see Table 6). No differences came to light for the control group. The trained group outperformed the control group at both post-test and follow-up (see Figure 1E). The net effect size for the trained group, adjusted on the control group's performance, was medium for both immediate gains (pre- vs. post-test) and long-term gains (see Table 6).

Figure 2E shows the values fitted from the best model for the trained group alone as a function of age, and of pre-test performance in the WM criterion task at the three assessment sessions, with the corresponding estimated linear trend. For age, it was the younger participants whose performance was better at post-test than at pre-test, with an evidence ratio of 443.44 (see Table 7), and they also maintained their better level of performance at follow-up, with an evidence ratio of 41.11 (see Table 7).

There was no differences in the slopes for pre-test performance in the WM criterion task, as confirmed by the evidence ratio. It is worth noting that there was a slightly higher variability in the Cattell test at post-test for participants with low scores for pre-test performance in the WM criterion task.

#### Processing speed

##### Pattern Comparison task

We considered a total of 121 models for the Pattern Comparison task. The best model was the one with the Session X CWMS baseline X Group X Vocabulary interaction, reaching a probability of 38.5%. This model was about two times more evident than the next, which reached a probability of around 20.5% (see Table 5).

For the Group (trained vs. control) X Session (pre-test vs. post-test vs. follow-up) interaction, the two groups did not differ at pre-test. The trained group performed better (taking less time to complete the task) at post-test than at pre-test, and maintained this improvement from post-test to follow-up (see Figure 1F). The effect sizes for group differences were large at post-test and became medium at follow-up (see Table 6). No differences were found for the control group. The trained group outperformed the control group at post-test and, to a certain extent at, at follow-up too (see Figure 1F). The net effect size for the trained group, adjusted on the control group's performance, was medium for both immediate gains (pre- vs. post-test) and long-term gains (see Table 6).

Figure 2F shows the values fitted from the best model for the trained group alone, as a function of vocabulary, and pre-test performance in the WM criterion task at the three assessment sessions, with the corresponding estimated linear trend. There was a very weak effect for vocabulary—the evidence ratio was 13.13 (see Table 7)—and it was the participants who had a higher pre-test vocabulary score who improved in the processing speed measure from pre- to post-test. No differences were found in the slope when the WM criterion task at pre-test was considered; a high individual variability also emerged (see Figure 2).

#### Inhibition

##### Stroop color task

We considered a total of 121 models for the Stroop color index on response times (RTs). The best model coincided with the Session X Age X Group interaction, with a probability of 22%, which is rather low, though this model was about 4 times more evident than the next one, which reached a probability of around 6.3% (see Table 5). As shown in Figure 1G, no Group (trained vs. control) X Session (pre-test vs. post-test vs. follow-up) interaction was found. This is in line with the null effect size identified on the differences between the groups, both immediately after the training and at follow-up (see Table 6), and also on the effect size computed for the immediate and long-term training gains obtained by the trained group (see Table 6).

The best model was only examined for the trained group. Figure 2G shows the values fitted from the best model as a function of age at the three assessment sessions, with the corresponding estimated linear trend. The results suggest that younger participants were more sensitive to interference, and that it decreased from pre-test to post-test, with an evidence ratio of 665.67 (see Table 7), but then rose again to the pre-test level at follow-up.

##### CWMS intrusions

We considered a total of 121 models for the CWMS intrusion errors. The best model was the one with the Session X Age X CWMS baseline X Group interaction, with a probability of 93%. This model showed a higher evidence than the others, since the associated probability was 18 times higher than that of the next model (see Table 5).

The differences were not very large for the Group (trained vs. control) X Session (pre-test vs. post-test vs. follow-up) interaction. Performance seemed to deteriorate from pre-test to post-test in the trained group, but not in the control group, while the number of errors increased at follow-up (see Figure 1H). The effect sizes for group differences were small at post-test and became medium at follow-up (see Table 6), but the two groups did not differ. The net effect size for the trained group, adjusted on the control group's performance, was small for immediate gains (pre- vs. post-test), but became medium for long-term gains (see Table 6).

Figure 2H the values fitted from the best model for the trained group alone as a function of age, and of pre-test performance in the WM criterion task, at the three assessment sessions, with the corresponding estimated linear trend. The regression slope for age decreased from post-test to follow-up, but not from pre-test to post-test, as supported by the evidence ratio, indicating that it was the older individuals who made more mistakes at pre-test, and it was only at follow-up that they were likely to experience fewer intrusion errors. The regression slopes for baseline performance in the WM criterion task decreased from session 0 (pre-test) to session 1 (post-test), with an evidence ratio of 799 (see Table 7), and then became flat, as supported by the evidence ratio (see Table 7): all the participants with a poor WM performance made more mistakes, in terms of intrusion errors, at pre-test, and fewer mistakes at post-test, and this improvement was maintained at follow-up.

## Discussion and conclusions

Our aim in the present study was to delineate how certain individual characteristics contribute to explaining WM training gains and transfer effects. Despite the importance of individual characteristics in cognition, only three studies in the aging literature have considered this issue. Here, age, formal education, general cognitive ability (operationalized with the vocabulary score), and WM baseline performance level were considered as a predictor of the short- and long-term specific training gains and transfer effects of a verbal WM training in a sample of healthy older adults.

To elucidate this issue, an analysis was conducted using linear generalized mixed effects models on data from four previous studies on healthy older adults that adopted the verbal WM training program developed by Borella et al. (2010). Part of the interest of such an analysis lies in that—for the first time, to our knowledge at least—all the studies examined were based on the same procedure and the same assessment measures, and they all included a follow-up session. The Borella et al. (2010) training program seems to be the only WM procedure to have been applied repeatedly in older adults with consistent results across studies. It is worth adding that another advantage of the studies selected for the present analysis is the inclusion of an active control group, and parallel versions of the tasks were presented (as recommended, but rarely done, in the literature; Zinke et al., 2012). The effects identified therefore cannot be attributed to the influence of item-specific practice.

Overall, our findings confirmed the efficacy of the verbal WM training procedure proposed by Borella et al. (2010): the trained group showed specific gains, performing better in the criterion task than the active control group immediately after the training, and maintaining this benefit at follow-up. Positive effects of the WM training were also generally apparent in terms of transfer effects, in the short term at least (at post-test), since the trained group outperformed the active controls in all the near transfer measures considered. As for the far transfer measures, the trained group again outperformed the active controls in all tasks, but not in terms of the Stroop Color index on RTs or CWMS intrusion errors.

This pattern of results was confirmed by the generally large post-test effect sizes (over 0.80) computed on the differences between the trained and active control groups, with the exception of the reasoning task –the Cattell test- (medium effect sizes) and intrusion errors in the CWMS (small effect sizes). At follow-up, the differences remained large for the criterion task, but became medium in the processing speed task—the Pattern Comparison task—and for intrusion errors in the CWMS, and small in the other tasks (Forward Digit and Backward Digit Span tasks, Dot Matrix task, and Cattell test). There were no changes in the effect size of the Stroop Color index on RTs, which confirms the absence of an effect of the training—the fact that there were no group differences—between the trained and control groups. Also by considering the net effect size of the training activities on participants' performance, that is changes in the trained group across sessions—pre-test, post-test and follow-up—(see Table 6) after adjusting the value for any change in the control group, the training benefits were confirmed. It is consequently reasonable to say that the training produced some maintenance effects on the trained group's performance.

These overall findings are consistent with the previously-published results obtained with the same WM training program. Although the present training regimen is quite short (only three training sessions) near and far training gains were found, confirming that this WM training procedure is effective. As also suggested by the meta-analysis conducted by Karbach and Verhaeghen (2014), the length of a training does not seem a crucial factor in determining its efficacy: in fact, most of the WM training procedures for older adults failed to document any benefits although they were much longer than the one considered here (see Borella et al., 2017; see also Table 1). The adaptive regimen adopted may well have favored training gains by: (i) ensuring that the tasks were always challenging, cognitively demanding and novel, consequently inducing participants to adhere to the task; (ii) producing a change in participants' allocation of attentional resources because the training engages several processes (including encoding, retaining information, inhibiting no longer relevant information, managing two tasks simultaneously, shifting attention, and attentional control) for an efficient handling of the different demands of the tasks. On the other hand, the lack of any short-term transfer effects for the two inhibitory measures may mean that inhibitory mechanisms are less amenable to training (see Zavagnin and Riboldi, 2012). Some degree of caution is required in interpreting the findings obtained with the Stroop Color task because they were based on RTs, which are not a very reliable indicator (e.g., de Ribaupierre et al., 2003; see Ludwig et al., 2010; Borella et al., 2017), and the sample was reduced for this particular measure (see Table 3). It is also possible, as discussed below, that it would take longer to prompt any detectable change for the inhibitory measures, or some of them at least.

Concerning long-term effects of the training, there was evidence of the maintenance of the specific training gain (in the criterion WM task), in line with all the WM training studies in the aging literature. In the transfer tasks, the training gains were only maintained for the Cattell test and the Pattern Comparison task, as seen in other studies using the same training procedure: the advantage of the trained group over the controls lay in the range of a medium effect size (or near-medium for the Pattern Comparison task). Such a selective maintenance of the training gains may be attributable to the well-documented strong relationship between WM and (i) processing speed (measured with the Pattern Comparison task), and (ii) reasoning ability (for a comprehensive discussion see Borella et al., 2010).

Improving WM performance makes cognitive operations more efficient, thus fostering the ability to move among the basic information processes. The other tasks may call upon more task-specific processes and abilities instead, leading to only transient (immediate) transfer effects (for a discussion, see Borella et al., 2010). One of the inhibitory measures examined, intrusion errors in the CWMS, seems particularly intriguing in that it only showed a clear improvement (fewer intrusion errors in the criterion task) at follow-up. This may mean that it would take longer to see a benefit of the training for some measures (inhibitory mechanisms in the present case). This phenomenon (i.e., clear transfer effects only at follow-up) has been found in other training studies in aging too (e.g., Borella et al., 2017), and has been called the “sleeper” effect. Although its nature needs to be further investigated (see Jaeggi et al., 2014), it may indicate that certain abilities take longer to show a significant improvement in performance. Future studies should make an effort to examine this issue.

Such a result on the intrusion errors and not in the Stroop Color task (leaving aside the problems associated with measuring RTs and the reduced sample size) may even indicate that WM training is more beneficial for some inhibitory functions than for others. In fact, intrusion errors in the CWMS and the Stroop Color task do belong to two different inhibitory functions. CWMS intrusion errors—an internal measure of the WM task and therefore closely related thereto (see Robert et al., 2009)—represent the resistance to proactive interference function of inhibition, which helps attention to be focused on relevant items and simultaneously-presented irrelevant items to be ignored; the Stroop Color task measures the resistance to prepotent response function of inhibition, which blocks dominant and prepotent cognitive responses automatically activated by the stimulus presented (e.g., Borella et al., in press). Resistance to proactive interference is also considered the only inhibitory function related to the control of information coming from memory content (Friedman and Miyake, 2004).

In light of the present findings, the questions following are: 1. Do any individual characteristics have a part to play in these findings? Are the effects of training supported by magnification or compensation effects, or both? Of course, there could be several aspects, such as methodological issues, but also participants' individual characteristics, capable of explaining the training gains and supporting the results. Here, we particularly analyzed the role of certain demographic variables (age and educational level), cognitive abilities in WM (i.e., pre-test performance in the criterion task), and a vocabulary test score as an indicator of crystallized intelligence.

Our findings showed that the role of the individual characteristics considered depended on the type of measure examined, and the effect of these variables was very modest for some tasks. The most interesting aspect seems to be that the factors considered would support either a compensation or a magnification effect immediately after the training, depending on which measure was analyzed. In particular, irrespective of the near or far transfer effects, the more the tasks demanded active information processing (i.e., the Dot Matrix, Backward Digit Span and Pattern Comparison tasks, the Cattell test, and the Stroop Color task), the more the factors examined seemed to support a magnification effect (of variable robustness). In other words, participants who had a higher initial performance in the crystallized measure used here and/or were younger, were more likely to improve after the training. For more passive tasks, on the other hand (i.e., the Forward Digit Span task, which is a short-term memory task), our results supported a compensation effect: participants with lower baseline vocabulary scores, an older age, and a weaker WM performance benefited more from the training. A particular pattern emerged for the criterion task (i.e., the task similar to the one used in the training) and for a closely related measure (CWMS intrusion errors): although the criterion task is complex, participants with a lower performance in a task of crystallized intelligence, as assessed with the vocabulary test, gained more from the training than those with higher vocabulary scores. That vocabulary should have such a role suggests that knowledge can counteract age-related decline (e.g., Baltes, 1987). Further, the role of vocabulary in explaining specific training gains may also suggest that participants exhibiting transfer effects were those who acquired new knowledge, rather than a greater processing efficiency. A finely-graded analysis at individual level may be able to clarify this issue.

As for intrusion errors there was evidence of a compensation effect related to age and baseline WM performance, with older participants and those with a lower baseline WM performance improving the most. These results may mean that the exercises used in the training enabled individuals with a lower crystallized ability to adapt to the demands of the tasks, engage better-controlled processes, and make more efficient use of their resources. It is hardly surprising that such a pattern of results should emerge with a training task that involves an adaptive procedure, which may have enabled progress to be made during the training, leading to a better performance and fewer intrusion errors at post-test and its maintenance at follow-up. Similarly, participants with a low baseline WM performance also became better able to manage no longer relevant information (CWMS intrusion errors).

One way to interpret these results could be by referring to the supply-demand mismatch conceptualized by Lövdén et al. (2010). According to these authors, changes in cognitive performance are induced by a mismatch between available resources and task demands: to cope with this mismatch, individuals engage in activities that promote flexibility, and consequently also plasticity. This hypothesis enables us to predict how individual differences might affect the benefits gained from a training regimen, depending on a task's complexity. The compensation effect seen for the criterion task (which closely resembled the task used in the training) may be due to the fact that using an adaptive procedure while practicing with the training task favored the “right amount” of supply-demand mismatch (i.e., demands exceeding than the available capacity) for individuals with a weaker profile in terms of their general cognitive abilities to re-activate their potential, and thus benefit from the training in terms of a better performance in the criterion task. It might have been easier to support this interpretation if our training procedure had been designed to enable us to test how performance changed from one training session to the next. Such an analysis would also shed light on what happens to individuals with the opposite profile (good general cognitive abilities—high vocabulary scores in the present study), who would only experience the mismatch if the WM tasks used in the training were more difficult, so instead of benefiting in terms of performance in the trained task they would gain in terms of plasticity in the transfer tasks.

According to the mismatch concept, the magnification effect found for the more demanding transfer tasks may indicate that in participants with a higher profile, in terms of age (i.e., younger individuals), or crystallized intelligence (i.e., those with higher vocabulary scores) the training induced a supply-demand mismatch that gave an impetus to change, thus engendering a flexible behavior. In participants with a lower profile, on the other hand, the high demands of the tasks used in the training might prevent any supply-demand mismatch because these individuals might abandon any attempt or be unable to apply resources and processes suited to the task.

The mixed results emerging from the present analysis as concerns the role of individual characteristics in explaining the compensation or magnification effects are consistent with a report from Zinke et al. (2013): the authors found that participants with weaker transfer effects were older (magnification), and that those with smaller training gains had stronger transfer effects (compensation). The role of age and WM performance varied, however, depending on the transfer tasks considered. The role of the predictors was examined too, but the effect size was small for some of the transfer tasks, and this limits the value of the results obtained.

It has to be said that the results found in the present study were modest too, so some degree of caution is warranted in interpreting them. Further, such a pattern of findings was found at post-test and, except for the criterion task and the intrusion errors, the role of the predictors was not maintained at follow-up for the other measures. Such a result could be interpreted in two different ways: one stems from on the idea that, because of the training, the individual characteristics are no longer significant because something beyond them has been modified during the training, such as the way in which participants process information; the other simply attributes the result to the fact that the effects of the training were not maintained. It therefore seems important to analyze the influence of other individual characteristics on the effects of WM training. This has been done, for the first time, at least to our knowledge, in both the short and the long term; the other three studies that approached such an issue did not consider the role of the predictors in the long term.

Our findings also suggest that compensatory and magnification effects are not mutually exclusive in explaining training gains; they may both contribute to characterizing and explaining the outcome of training. It would therefore be important for future WM training studies in general (and in aging, in particular) to make the effort to examine the role of individual factors. We are aware that large samples are needed for such analyses, but it is only by trying to overcome such practical problems that research can advance and enable us to ascertain the real usefulness of intervention to promote an active aging.

A number of limitations of the present study have to be acknowledged. First, single measures were used to represent the constructs of interest, whereas using multiple indicators of the same process (e.g., Shipstead et al., 2010) in training studies would enable us to draw stronger conclusions. Second, we were unable to consider training gains *per-se*, i.e., the role of individual differences in improvements induced by training across the sessions, the rate of learning, in predicting the outcome of the training, because the particular procedure used did not allow for these aspects to be analyzed. Examining what progress a given individual makes, and how it relates to the effects of the training could shed further light on the value of the present training program (Zinke et al., 2013; Bürki et al., 2014). A third limitation lies in that we analyzed the role of a limited number of factors potentially influencing training gains. In future studies we plant to conduct a more complete assessment of general cognitive ability, and assessing them with tasks not used to test transfer effects (to avoid multi-collinearity problems). Since we acknowledge the exploratory nature of our results, we have discussed overall trends for the transfer tasks, rather than the influence of each of the specific predictors on each task. There are also other factors, of course, that may have influenced the training gains, and that have yet to be considered, such as metacognitive (motivational) variables, mood, and psychological well-being (e.g., von Bastian and Oberauer, 2014; Könen and Karbach, 2015). It might also be of interest to analyze the influence of genetics (such as dopamine availability) on training gains (von Bastian and Oberauer, 2014). Future studies should therefore strive to include a broad array of factors, with larger and more homogeneous samples (i.e., with same size) than the one used here, in an effort to delineate all the conditions capable of shaping the effects of WM training in older adults.

To conclude, the present study provides further evidence of the elderly gaining in cognitive flexibility and plasticity from a verbal WM training. It also highlights the importance of analyzing the factors influencing WM training gains in aging. Also, although we showed that older people's WM can be improved thanks to a plasticity that persists with aging, we found that the role of individual characteristics depended on the transfer measure examined. It is consequently important to ascertain “who” gains from the training, but also “who gains in which tasks,” in order to be able to design the most effective WM training to suit an individual's cognitive profile. This study could thus be considered as one of the first promising steps toward clarifying the impact of individual characteristics on the short- and long-term efficacy of WM training.

## Author contributions

EB designed the study, assisted in carrying out the analyses, and wrote the paper. EC wrote the paper and assisted in carrying out the analyses. MP carried out the statistical analyses. RD assisted in writing the paper. BC designed the study, assisted in carrying out the analyses, and wrote the paper.

## Funding

The study was supported by the grant CPDA141092/14 awarded by the University of Padova to BC.

### Conflict of interest statement

The authors declare that the research was conducted in the absence of any commercial or financial relationships that could be construed as a potential conflict of interest. The reviewer JMR and handling Editor declared their shared affiliation, and the handling Editor states that the process nevertheless met the standards of a fair and objective review

## Footnotes

^1^As for the Forward and the Backward Digit Span tasks and the Pattern Comparison task, these measures were used in three (Borella et al., 2010, 2013, 2017) of the four studies considered. The pooled trained (*n* = 56) and control (*n* = 56) groups from these three studies did not differ in terms of age (trained group: *M* = 72.43 *SD* = 5.81; control group: *M* = 72.04, *SD* = 6.16), *F*_(1, 110)_ < 1, years of formal education (trained group: *M* = 9.482 *SD* = 4.72; control group: *M* = 10.30 *SD* = 4.65), *F*_(1, 110)_ < 1, or vocabulary score on the Wechsler Adult Intelligence Scale - Revised (WAIS–R; Wechsler, 1981; trained group: *M* = 50.23 *SD* = 11.27; control group: *M* = 49.07 *SD* = 11.45), *F*_(1, 110)_ < 1. As for the Cattell test, this was used in three (Borella et al., 2010, 2013; Carretti et al., 2013b) of the four studies considered. The pooled trained (*n* = 55) and control (*n* = 57) groups from these two studies did not differ in terms of age (trained group: *M* = 72.35, *SD* = 5.86; control group: *M* = 72.72 *SD* = 5.47), *F*_(1, 110)_ < 1, years of formal education (trained group: *M* = 7.80 *SD* = 3.73; control group: *M* = 8.49 *SD* = 4.07), *F*_(1, 110)_ < 1, or vocabulary score on the Wechsler Adult Intelligence Scale—Revised (WAIS–R; Wechsler, 1981; trained group: *M* = 45.11 *SD* = 8.89; control group: *M* = 43.16 *SD* = 10.25), *F*_(1, 110)_ = 1.15, *p* = 0.28. The Dot Matrix task and the Stroop Color task were used in two (Borella et al., 2010, 2013) of the four studies considered. The pooled trained (*n* = 38) and control (*n* = 38) groups from these two studies did not differ in terms of age (trained group: *M* = 73.84, *SD* = 6.12; control group: *M* = 73.89 *SD* = 5.85), *F*_(1, 74)_ < 1, years of formal education (trained group: *M* = 7.66 *SD* = 3.79; control group: *M* = 8.24 *SD* = 3.62), *F*_(1, 74)_ < 1, or vocabulary score on the Wechsler Adult Intelligence Scale—Revised (WAIS–R; Wechsler, 1981; trained group: *M* = 44.79 *SD* = 8.93; control group: *M* = 44.21 *SD* = 9.65), *F*_(1, 74)_ <1.

^2^For the CWMS, the baseline performance in the CWMS was not entered as a predictor because of multicollinearity problems.
